# Resistance Exercise Intensity is Correlated with Attenuation of HbA1c and Insulin in Patients with Type 2 Diabetes: A Systematic Review and Meta-Analysis

**DOI:** 10.3390/ijerph16010140

**Published:** 2019-01-07

**Authors:** Yubo Liu, Weibing Ye, Qian Chen, Yong Zhang, Chia-Hua Kuo, Mallikarjuna Korivi

**Affiliations:** 1 Exercise and Metabolism Research Center, College of Physical Education and Health Sciences, Zhejiang Normal University, Jinhua 321004, China; liuyubo0124@outlook.com (Y.L.); zhangyong@zjnu.cn (Y.Z.); 2 Zhejiang Sports Science Institute, Hangzhou 310004, China; cq_chenqian@hotmail.com; 3 Department of Sports Sciences, University of Taipei, Taipei 111, Taiwan; kuochiahua@gmail.com

**Keywords:** diabetes, strength training, glycosylated hemoglobin, insulin, meta-regression

## Abstract

We investigated the influence of resistance exercise (RE) with different intensities on HbA1c, insulin and blood glucose levels in patients with type 2 diabetes (T2D). Diabetes trials that compared RE group with a control were included in meta-analysis. Exercise intensities were categorized into low-to-moderate-intensity and high-intensity subgroups. Intensity effect on glycemic control was determined by meta-regression analysis, and risk-of-bias was assessed using Cochrane Collaboration tool. 24 trials met the inclusion criteria, comprised of 962 patients of exercise (*n* = 491) and control (*n* = 471). Meta-regression analysis showed decreased HbA1c (*p* = 0.006) and insulin (*p* = 0.015) after RE was correlated with intensity. Subgroup analysis revealed decreased HbA1c was greater with high intensity (−0.61; 95% CI −0.90, −0.33) than low-to-moderate intensity (−0.23; 95% CI −0.41, −0.05). Insulin levels were significantly decreased only with high intensity (−4.60; 95% CI −7.53, −1.67), not with low-to-moderate intensity (0.07; 95% CI −3.28, 3.42). Notably, values between the subgroups were statistically significant for both HbA1c (*p* = 0.03) and insulin (*p* = 0.04), indicative of profound benefits of high-intensity RE. Pooled outcomes of 15 trials showed only a decreased trend in blood glucose with RE (*p* = 0.09), and this tendency was not associated with intensity. Our meta-analysis provides additional evidence that high-intensity RE has greater beneficial effects than low-to-moderate-intensity in attenuation of HbA1c and insulin in T2D patients.

## 1. Introduction

Type 2 diabetes (T2D) is the most common type (90%) of diabetes, characterized by hyperglycemia in the context of insulin resistance and impaired insulin secretion [1,2]. Typically T2D is accompanied by a cluster of risk factors, including dyslipidemia, hypertension, and cardiovascular diseases, therefore causing a severe financial burden on the global health care system [3,4]. According to the latest statistics from the International Diabetes Association (IDF, 2017), the incidence of diabetes in adults (20–79 years) has risen abruptly to 425 million worldwide, and this number is projected to increase to 629 million by 2045. Currently, the largest number of people with diabetes (20–79 years) are in China (114 million), India (73 million), and in the USA (30 million) [5]. Not surprisingly, sedentary lifestyle, unhealthy diet, and urbanization are strongly associated with the prevalence of T2D in adults [5,6]. In this perspective, IDF and several randomized controlled trials (RCTs) have emphasized that lifestyle modification with good physical activity and/or healthy diet can delay or prevent the onset of T2D [1,7,8,9].

Physical activity, especially aerobic exercise (AE) has consistently been reported to ameliorate the glycemic control, insulin resistance and dyslipidemia in patients with T2D [10,11]. Despite conventional recommendation of AE, recent findings have revealed the importance of resistance exercise (RE) in efficient management of diabetes [2,12,13]. The American College of Sports Medicine (ACSM) also recommended incorporation of progressive RE to treat T2D [14]. A joint position statement by ACSM and the American Diabetes Association (ADA) claimed that both resistance and aerobic training can improve insulin action, and assist in management of blood glucose, lipids, cardiovascular risk factors, and quality of life [11]. However, a RCT reported that 26-week RE, but not AE significantly lowers the remnant-like lipoprotein cholesterol in patients with T2D [12]. A systematic review and meta-analysis indicated that both RE and AE are effective in controlling the diabetes (decreased glycosylated hemoglobin (HbA1c)), and there is no evidence that RE differs from AE on cardiovascular risk factors or safety [10]. Increasing popularity of resistance training in recent decades could be attributed to its promising health promotion benefits in the diabetic population. Therefore, it is worthwhile to explore further details on the influence of RE and RE variables, and whether they are responsible for such beneficial effects in controlling T2D.

On the other hand, aerobic training, including jogging, brisk walking, cycling, and swimming recruits a large group of muscles to perform, and usually requires prolonged periods [10,15]. In this context, it is infeasible to achieve the required volume and intensity of the AE to control T2D, as most patients with T2D were obese or overweight with mobility problems. Additionally, T2D is often accompanied with physical disability, visible impairments or cardiovascular burdens [16,17]. Given that, RE, which uses muscular strength to move a weight or to work against a resistive load, causing isolated, brief activity of single muscle groups might be a more feasible approach to achieve the goal without additional difficulties. For instance, resistance training reported to promote insulin sensitivity via increased muscle mass, glucose uptake, and facilitate glucose clearance from the circulation [11,18]. High-intensity progressive resistance training (PRT, 75–85% 1-repetition maximum (1RM)) has been shown to be safe for older diabetic patients, and improved glycemic control (decreased HbA1c) and muscle strength [15]. Furthermore, RE can be performed in a residential setting and is more appropriate for sedentary, elderly T2D patients with worse muscle strength [10].

Although RE is reported to be safe and effective in the management of T2D, the influence of different RE intensities on changes in HbA1c, insulin, and blood glucose levels remains to be elucidated in patients with T2D. The existing literature reveals equivocal results of RE with different intensities on modulating the diabetes biomarkers, when prescribed to the diabetic population [15,19,20]. Additionally, no meta-analysis has compared the effects of different RE intensities on glycemic control in patients with T2D. Based on RE intensity, we categorized the trials into low-to-moderate-intensity and high-intensity subgroups and evaluated whether intensity is associated with its beneficial effects. The data collected from all available sources were included into meta-analysis and examined effective intensity of RE in controlling the HbA1c, insulin, and blood glucose concentrations in patients with T2D.

## 2. Materials and Methods

### 2.1. Data Sources and Searches

We conducted a literature search using electronic databases, including PubMed, SportDiscus, ScienceDirect/Scopus, Google Scholar, EMBASE, and WanFang. The articles published in English until September 2018 were searched and collected using the following keywords: ‘resistance exercise’ OR ‘strength exercise’ OR ‘resistance training’ OR ‘strength training’ with combination of ‘type 2 diabetes’ or ‘T2D’. Each exercise/training keyword was independently used with ‘type 2 diabetes’ or ‘T2D’, and search was performed separately. An additional search was also done from the reference list of some selected articles, and included for the analysis.

### 2.2. Inclusion and Exclusion Criteria

Prior to inclusion, titles and abstracts of the searched articles were screened for relevance. Then full-text of the articles were obtained and reviewed for the inclusion criteria. To include the articles, we have followed these inclusion criteria: (1) All individuals were patients with definite T2D; (2) studies were randomized controlled trials (RCT) published in English; (3) the duration of resistance exercise (RE) was 6 weeks or more and performed alone, not combined with aerobic exercise (AE); (4) all intervention measures taken by the control group were same as the RE group, except exercise; and (5) all studies provided mean values of diabetic indices before and after RE intervention. We excluded studies according to these criteria: (1) clinical trials without control or studies dealing with animals; (2) studies that did not measure fasting blood glucose or blood glucose 2 h after meal (postprandial) were excluded; (3) the control trial was not diabetes, and the study purpose was not to control the blood glucose, HbA1c, or insulin levels in patients; (4) research papers of repeated reports, poor quality or insufficient information about RE; (5) papers with poor basal equilibrium or diverse baseline of blood glucose or HbA1c values.

Article search, data collection, and evaluation were performed by two authors (YL and WY) independently. The other review authors (YZ, QC, and CHK) provided additional review and insight. Any disagreements on inclusion or exclusion of trails into the study were discussed and confirmed by another review author (MK).

### 2.3. Data Extraction

**Figure 1:**
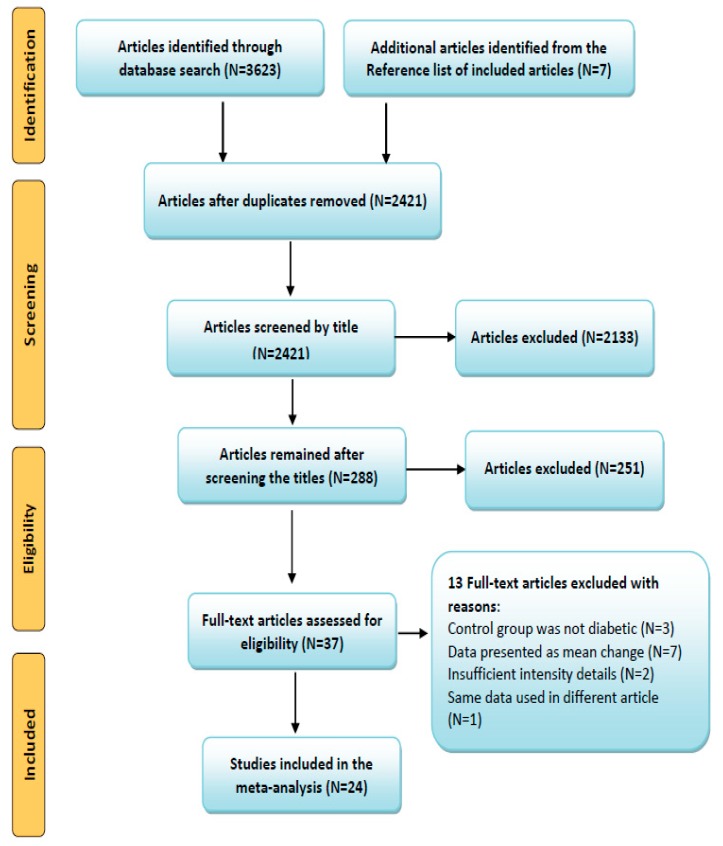
Preferred Reporting Items for Systematic Review and Meta-analysis (PRISMA) flow diagram of study selection.

A total of 3623 articles were retrieved from the databases, and 24 articles consisting of 962 participants (exercise 491, control 471) were included in this meta-analysis according to the inclusion criteria. The detailed selection process of the article was documented in a Preferred Reporting Items for Systematic Reviews and Meta-Analyses (PRISMA) flow diagram (Figure 1). Information on the selected articles, including publishing year, participants’ age, sex, diabetes duration and details of RE (intensity, frequency, duration) were tabulated, and presented in Table 1. Data from the selected articles were extracted by three independent review authors (YL, WY, and MK), and presented as mean and standard deviation (SD). Standard errors provided in those studies were converted to SD.

### 2.4. Risk of Bias Assessment

The Cochrane Collaboration tool was used to determine the risk of bias [21]. Included full-text articles were assessed by two of the three review authors (YL, WY, and YZ), and applied the risk of bias tool independently to each study. The differences were resolved by discussing with another review author (MK). The source of bias, such as selection bias (random sequence generation and allocation concealment), performance bias (blinding of participants and personnel), detection bias (blinding of outcome assessment), attrition bias (incomplete outcome data) and reporting bias (selective reporting) were detected for the included trials. The detailed outcome of the risk of bias was summarized in the results section.

### 2.5. Subgroup Division and Observed Indices

Based on the intensity, included trials with RE were categorized into two subgroups, including low-to-moderate-intensity and high-intensity trials. The intensity between 20% and 75% 1RM considered as low-to-moderate- and intensity between 75% and 100% 1RM considered as high-intensity RE. This subgroup category was followed according to the guidelines described in ACSM’s Foundations of Strength Training and Conditioning [22]. The changes in key biomarkers in T2D, such as fasting blood glucose, insulin, and glycosylated hemoglobin (HbA1c) were included for the meta-analysis.

### 2.6. Statistical Analyses

The data analysis was performed using statistical software of the Cochrane Collaboration Review Manager (RevMan, version 5.2, Copenhagen, Denmark). The main statistical procedures include heterogeneity analysis, computation, and verification of combined effect size. The fixed effect model was used for meta-analysis if no significant difference was found in heterogeneity analysis (*p* > 0.05). The random effect model was used if heterogeneity was found significant (*p* < 0.05). Upon heterogeneity significance (pooled outcome), we performed meta-regression analysis to examine the association between variables of RE (intensity (% 1RM), frequency, sets, and duration) and changes in diabetic biomarkers (HbA1c, insulin, and blood glucose levels). For measurement data, weighted mean difference (MD) was used and expressed as a 95% confidence interval (95% CI). For the meta-regression analysis, we used STATA version 12 (StataCorp, College Station, TX, USA). The changes in HbA1c and insulin levels after RE were identified to be correlated with exercise intensity variable. Therefore, we categorized the trials into two subgroups, low-to-moderate-intensity and high-intensity to identify the effective intensity of RE. The differences between the subgroups (intensities) was also analyzed and indicated as a significant difference.

## 3. Results

### 3.1. Search Results and Article Selection

Through the systematic search, we identified a total of 3623 (+7) articles from all databases, and initially excluded 1209 duplicates. After screening the titles of the rest of the 2421 articles, 288 were selected for the abstract and full-text assessment, and 37 of them were included in this study, which met the required inclusion criteria. Out of 37, 13 articles were excluded with the following reasons: control group was not diabetic in three trials [23,24,25], data presented as mean difference or no comparable diabetic control trial in seven studies [13,15,23,26,27,28,29], two articles with insufficient exercise intensity details [30,31] and same data used in different articles [32]. Finally, 24 articles [19,20,33,34,35,36,37,38,39,40,41,42,43,44,45,46,47,48,49,50,51,52,53,54] were included in the meta-analysis. Article selection was done according to the PRISMA guidelines. The comprehensive steps of the selection process and number of articles in each step were presented as a flow diagram in Figure 1.

### 3.2. Description of the Included Articles

In this meta-analysis of 24 trials, total 962 patients with T2D were enrolled (491 exercise, 471 control). The characteristics of patients and RE details were presented in Table 1. Briefly, the selected studies were intercontinental, including from Australia, Brazil, Canada, China, England, Finland, Germany, Grease, India, Iran, Japan, New Zealand, South Korea, and USA. The included trials according to inclusion criteria, were published between 1997 and 2018. Among them, three trials recruited only female patients, three studies recruited only males, 14 trials were a combination of both, and no gender information for four trials. The mean age of patients was between 45 and 71 years, and their baseline HbA1c was 7.7% and 7.27% in control and exercise trails, respectively (after intervention). According to the data from the trials, the duration of diabetes ranged from more than half a year to 13 years, and the duration of RE performance was ranged from 6 to 52 weeks (Table 1).

### 3.3. High-Intensity RE Prominently Reduces HbA1c Than Low-To-Moderate-Intensity in Patients with T2D

**Figure 2:**
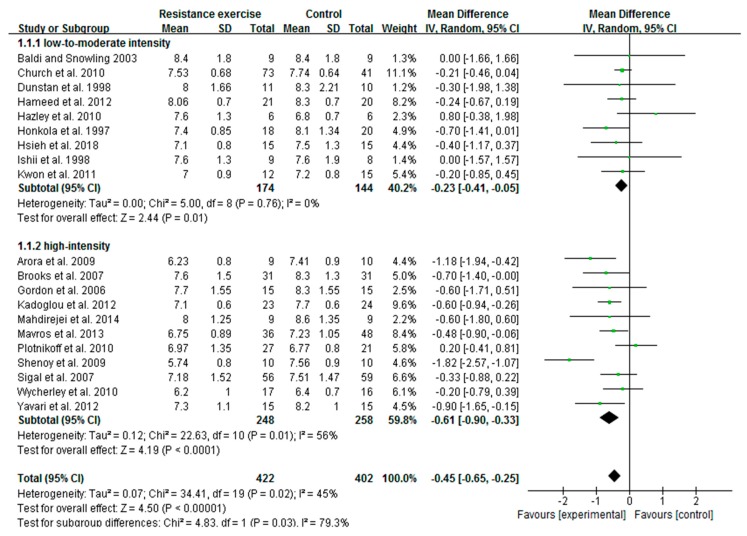
Forest plot of HbA1c changes with different intensities of resistance exercise in patients with type 2 diabetes. SD, standard deviation; IV, inverse variation; CI, confidence internal; df, degrees of freedom.

Of 24 included articles, 20 studies measured HbA1c as an index of glycemic control in patients with T2D. A total of 824 patients, including 422 from exercise and 402 from control trials completed the study. The pooled outcome showed that the change in HbA1c was extremely favored to exercise intervention with heterogeneity Tau^2^ = 0.07; Chi^2^ = 34.41; df = 19 and I^2^ = 45%. Meta-regression analysis revealed the exercise intensity variable is correlated with the changes of HbA1c. Based on RE intensity, we then assigned 20 trials into low-to-moderate-intensity (9 articles) and high-intensity (11 articles) subgroups, and the influence of intensity on HbA1c change was evaluated. We found both low-to-moderate-intensity (MD = −0.23; I^2^ = 0%; 95% CI: −0.41 to −0.05, *p* = 0.01) and high-intensity (MD = −0.61; I^2^ = 56%, 95% CI: −0.90 to −0.33, *p* = 0.0001) RE substantially decreased the HbA1c levels in diabetic patients. However, the decreased HbA1c with high intensity was more prominent than that of low-to-moderate-intensity exercise. Further, the differences between subgroups reached statistical significance (*p* = 0.03) with greater reduction in the high-intensity subgroup (Figure 2). These findings revealed that the beneficial effect of RE is associated with its intensity in reduction of HbA1c levels.

### 3.4. High-Intensity, Not Low-To-Moderate-Intensity RE Decreases Insulin Levels

**Figure 3:**
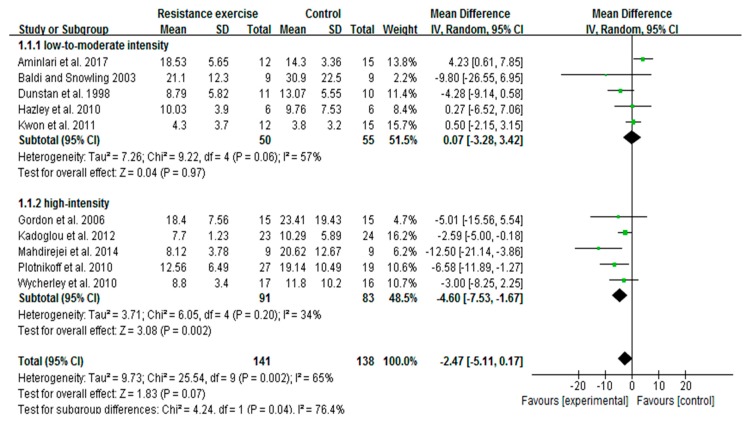
Forest plot of insulin changes with different intensities of resistance exercise in patients with type 2 diabetes. SD, standard deviation; IV, inverse variation; CI, confidence internal; df, degrees of freedom.

We extended our analyses to find out whether RE intensity is correlated with changes of insulin levels in diabetic patients. A total of 10 trials (279 participants) with fasting insulin data were included for the meta-analysis. Initial pooled outcome showed the overall decrease of insulin with RE (irrespective of intensities) was marginal in patients (*p* = 0.07). We conducted meta-regression analysis, and noticed the decreased trend of insulin was associated with exercise intensity. Subsequent subgroup analysis was carried out to identify the effective RE intensity on insulin changes. The findings revealed that high-intensity trials [33,37,39,41,53] were represented by a remarkable decrease of insulin (MD = −4.60; I^2^ = 34%; 95% CI: −7.53 to −1.67; *p* = 0.002), while trials with low-to-moderate intensity [20,34,35,44,45] did not show a significant decrease of insulin (MD = 0.07; I^2^ = 57%; 95% CI: −3,28 to 3.42, *p* = 0.97). Interestingly, test results for subgroup differences were significant between the subgroups (*p* = 0.04), which emphasizes the correlation between RE intensity and degree of insulin change (Figure 3).

### 3.5. RE Trends to Decrease Blood Glucose Levels in Patients with T2D

**Figure 4:**
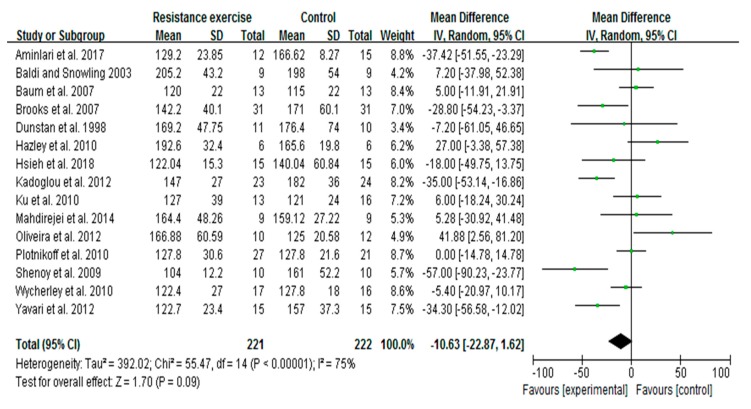
Pooled outcome of changes in blood glucose levels after resistance exercise in patients with type 2 diabetes. SD, standard deviation; IV, inverse variation; CI, confidence internal; df, degrees of freedom.

In this meta-analysis, a total of 15 studies of 443 patients with fasting blood glucose data were included to determine the effect of RE on alterations in blood glucose levels. Irrespective of RE intensity, pooled outcome showed that RE slightly decreased the blood glucose levels in patients with T2D. The overall mean difference was −10.63 with I^2^ = 75%; 95% CI: −22.87 to 1.62, and the *p* = 0.09 (Figure 4). Further, to examine whether the intensity variable is correlated with this tendency, we performed meta-regression analysis for these 15 trials. We found that the decreased tendency of blood glucose with RE was not associated with the exercise intensity variable (*p* = 0.39). Results from subgroup analysis showed no statistical difference with low-to-moderate-intensity (*p* = 0.67) or high-intensity (*p* = 0.09) RE, and no difference between the subgroups (*p* = 0.59) (data not shown).

### 3.6. Summary of Risk of Bias

**Figure 5:**
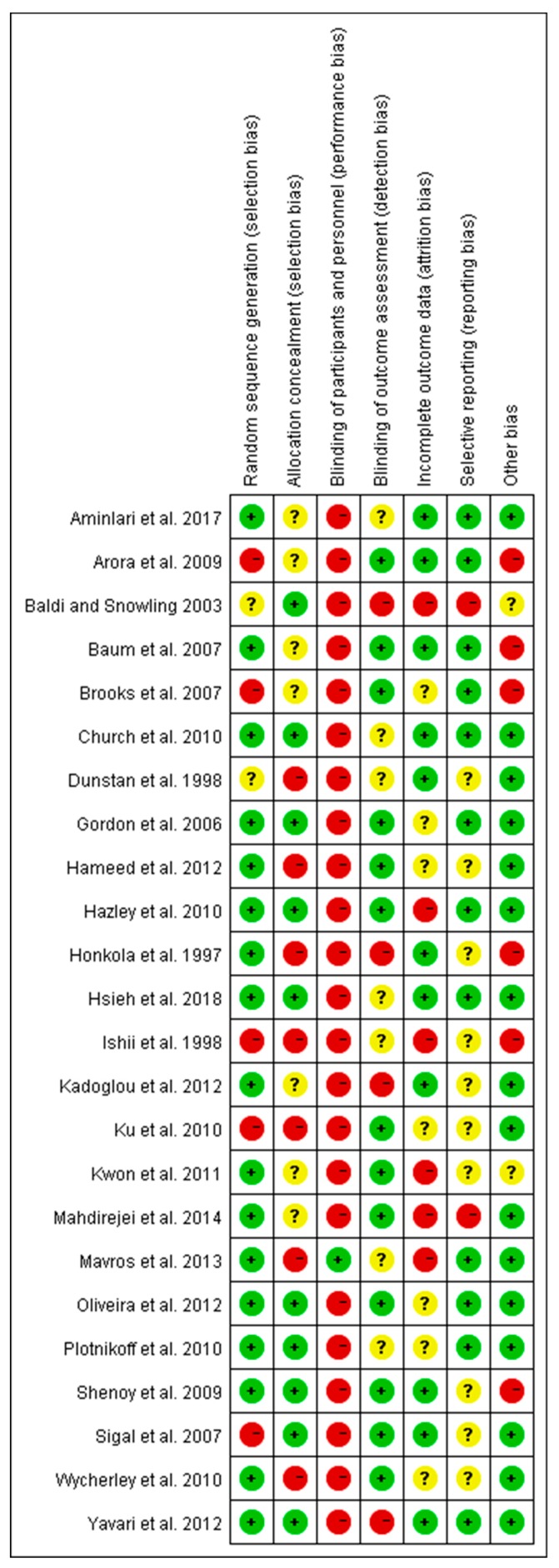
Summary of the risk of bias for the trials included in this meta-analysis. Green indicates low risk of bias, yellow indicates unclear, and red indicates high risk of bias.

Risk of bias in this study was assessed using the Cochrane Collaboration method, and the detailed statement was presented in Figure 5. For the selection bias, only five trials reported random sequence generation [19,36,38,40,47], and seven trials reported allocation concealment [19,33,34,47,48,51,52]. For the performance bias, except for one trial [51], all trials judged to have high risk of bias for blinding patients towards RE intervention. The study by Movros and colleagues [51] adopted a sham group. In most cases, it may not be possible to blind the participants in an exercise intervention. However, reporting such high risk of bias did not necessarily compromise the quality of the study. Instead, other variables, including the level of study attrition, poor intervention adherence, and selective reporting bias are the most common issues around the high risk of bias that would impact on study quality [55]. In our assessment, only two trials were identified with reporting bias [37,44]. Four trials appeared to have detection bias [41,44,46,48], and six articles reported to have attrition bias [19,20,35,37,44,51]. In this analysis, the highest number studies (17 trials) were found to have a low risk of bias for the random sequence generation.

## 4. Discussion

To the best of our knowledge, this is the first meta-analysis and systematic review to compare the effect of two different intensities of RE on HbA1c, insulin, and blood glucose levels in patients with T2D. We demonstrated that the decreased HbA1c and insulin (not blood glucose) values with RE were associated with its intensity in diabetic patients. We further identified that both high- and low-to-moderate-intensities substantially reduced HbA1c. However, for insulin, only high intensity contributed to a significant reduction, while low-to-moderate intensity had no effect. On the other hand, pooled outcome of 15 trials showed only a marginal decrease of blood glucose with RE (irrespective of intensity), and this tendency was not associated with RE intensity, unlike HbA1c and insulin. Taken together, our meta-analysis revealed that high-intensity RE has greater beneficial effects than low-to-moderate-intensity in decreasing the HbA1c and insulin levels in patients with T2D. Despite the ACSM and ADA guidelines to include RE as part of a well-rounded program for the effective management of diabetes [11], RE intensity appears to be the primary concern to accomplish the goal.

It has been indicated that manipulation of exercise variables, such as intensity, duration, volume, or frequency may optimize the glucose-lowering effect in different population [56,57]. Therefore, it would be interesting and useful to understand which variable is associated with greater beneficial effects of exercise in patients with T2D. In this meta-analysis and systematic review, we focused on the intensity variable of RE that could control HbA1c, insulin, and blood glucose levels in diabetic patients. HbA1c is a key determinant for the risk of diabetes-associated complications and mortality. However, effective management of HbA1c levels in diabetic patients could reduce this burden [58]. It has been well documented that each 1% decrease in HbA1c value was associated with 14% reduction of myocardial infarctions and 21% decrease of risk-of-death related to diabetes [59]. Another study reported lowering HbA1c in patients with T2D decreases the risk of developing coronary heart disease by 5–17%, and all-cause mortality by 6–15% [60]. In a target to treat T2D, mounting evidence demonstrated that RE, irrespective of its intensity effectively decreases the HbA1c levels in patients, and thereby prevents diabetes-associated complications [13,15,56,61]. A RCT from Italy showed both resistance and aerobic trainings lowered the HbA1c to a similar extent by 0.35% and 0.40% respectively, in subjects with T2D [26]. In contrast, another RCT from Vienna emphasized that only strength training decreased the HbA1c (1.2%), not endurance training in T2D patients [27]. Findings from an Australian RCT addressed that high-intensity PRT with moderate weight loss considerably decreased HbA1c at 3 months (0.6%) and 6 months (1.2%) in older diabetic patients [15]. Another interesting trial from India demonstrated that moderate-intensity PRT for 3 months significantly decreased HbA1c levels (0.54%) in Asian Indians with T2D [13]. Despite the existing reports on RE-induced HbA1c reduction, the comparable association between RE intensities and degree of HbA1c reduction has not been elucidated in diabetic patients.

To the best of our knowledge, this is the first report to compare two training intensities of RE on HbA1c change in diabetic patients. In this meta-analysis (20 trials), we found both high-intensity and low-to-moderate-intensity RE significantly decreased the HbA1c. The greater reduction of HbA1c was found with high-intensity RE compared to low-to-moderate-intensity. In contrast, a meta-analysis of eight studies in 2011 concluded that resistance training alone had no significant effect on HbA1c levels in diabetic patients. These eight studies involved three supervised exercise sessions per week with intensity ranging between 50% and 80% 1RM [62]. A systematic review and meta-analysis of 14 RCTs displayed that AE training might be more efficient than resistance training in decreasing HbA1c and fasting blood glucose levels in patients with T2D. Nonetheless, these findings could not be affirmed when included only low risk of bias trials into the analysis, and exercise was performed under supervision [61]. Another meta-analysis stated both RE and AE decreases HbA1c, and there is no evidence to claim that RE is different from AE in cardiovascular risk factors or safety [10].

To address the association between intensity/volume of exercise training (aerobic, resistance, or combined) and HbA1c changes in patients with T2D, Umpierre et al. conducted a systematic review with meta-regression analysis of 26 RCTs. They found that changes in HbA1c were not correlated with any variable (intensity or volume) of RE, whereas in AE training, changes in HbA1c were associated with exercise volume [56]. In this line, a recent meta-analysis (eight trials) indicated that high-intensity RE tend to decrease HbA1c more than low-intensity RE in T2D patients, and other variables, including duration, frequency, and volume appear to be ineffective [2]. Moderate-intensity (40–50%) high-volume resistance training had no effect on HbA1c levels in diabetic subjects [19]. Based on the duration of RE, a recent meta-analysis categorized seven trials into two subgroups, 8–20 weeks (four trials) and 21–48 weeks (three trials), and found no differences in HbA1c between the subgroups and also with overall RE [63]. None of these studies categorized RE based on its intensity, and compared intensity effects on change of HbA1c in diabetic patients. Our meta-analysis showed 0.61% and 0.23% reduction of HbA1c with high- and low-to-moderate-intensity RE, respectively. This strong correlation between RE intensity and HbA1c reduction suggests that high-intensity RE may be a suitable approach to control the elevated HbA1c levels in T2D patients.

Another important finding of our meta-analysis is that an RE-induced insulin decrease was seen only in the high-intensity subgroup, not in the low-to-moderate-intensity subgroup. Moreover, the difference between the subgroups was statistically significant, which represents the strong influence of high intensity in controlling the insulin response of diabetic patients. Of note, several RCTs on a diabetic population reported decreased or unchanged insulin levels after RE, without discussing the precise influence of exercise intensity. A study on diabetic patients by Ishii et al. reported improved insulin sensitivity with moderate-intensity (40–50% 1RM) high-volume resistance training, however, no possible reasons behind this increase were explained [19]. In contrast, plasma insulin levels were found to remain the same even after 6-month high-intensity (75–85% 1RM) PRT in older diabetic patients [15]. A study on older T2D patients concluded that 16-week resistance training (50–80% 1RM) significantly improved insulin sensitivity (46.3%), increased muscle strength, and decreased abdominal fat, however, HbA1c levels remained unchanged [64]. The discrepancy results of RE on insulin sensitivity are possibly due to varied or inadequate intensities of exercise, physical fitness of patients, and/or differences in methods to measure the insulin sensitivity.

Increased insulin sensitivity after RE has been shown to be associated with a concomitant decrease of visceral and abdominal subcutaneous adiposity or abdominal obesity [65]. A cohort study on diabetic subjects found significantly improved insulin resistance (~15%), metabolic features, and reduced abdominal fat after 4-month resistance training (70–80% 1RM), and this phenomenon was similar to the aerobic training [26]. Four months strength training (up to 85% 1RM) reported improved insulin sensitivity and lipid profile in diabetic patients, while the endurance training effect was moderate [27]. Moderate-intensity RE for 3-months improved insulin sensitivity along with decreased subcutaneous adipose tissue in Asian Indian diabetic patients [13]. Improved insulin sensitivity with RE perhaps occurs without increasing the muscle mass [13], through increased skeletal muscle GLUT4 protein expression and insulin signaling [66]. At a molecular level, GLUT4 is stimulated upon muscle contraction and/or insulin, which primarily transport glucose to other tissues of the body. Increased skeletal muscle GLUT4 protein following strength training has been described as a possible reason behind the enhanced insulin action in patients with T2D [67].

Our study pointed out that decreased blood glucose with RE (irrespective of intensity) is not convincing like HbA1c and insulin reductions in patients with T2D. It appears that the beneficial effects of RE on glycemic biomarkers (HbA1c, insulin, and blood glucose) does not occur in a similar fashion. This might be due to the involvement of specific factors or mechanisms that regulate each glycemic biomarker in patients after exercise intervention. The existing RCTs of diabetic patients also witnessed for the divergent effects of RE on changes in blood glucose levels. For instance, high-intensity RE (75–85% 1RM) had no effect on blood glucose levels at 3- and 6-months after training in older diabetic patients [15]. On the other hand, moderate-intensity resistance training (3-month) significantly decreased fasting blood glucose in Asian Indians with T2D [13]. In contrast, 8-week moderate-intensity RE (50–60% 1RM) reported to be ineffective in reducing the blood glucose in diabetic patients [20]. The existing studies of RE on the diabetic population have demonstrated a positive effect on one or more metabolic risk factors, such as HbA1c, insulin sensitivity, fasting blood glucose, or lipid profile. In those studies, RE programs have been greater than 8 weeks, at least 3-sessions per week with a high intensity of 60–80% 1RM [15]. A recent meta-analysis recommended that older patients with T2D need to pay more attention to the intensity of RE rather than duration, frequency, or volume to improve the glycemic control [2]. Taken together, the extent of blood glucose changes with RE is considerably diverse, and therefore, the concrete effect of RE intensity on blood glucose levels alone is perhaps inconclusive in diabetic patients.

### Significance of Resistance Exercise at Molecular Level

Resistance training-induced physiological stimuli and/or specific molecular signaling cascades can facilitate a number of physiological adaptations in individuals, and thereby mitigate the diabetes complications. For instance, RE induces beneficial changes in insulin sensitivity through increased skeletal muscle mass, glucose storage, enhanced glucose clearance from circulation, and improved mitochondrial oxidative capacity [3,61]. Improved insulin sensitivity in T2D was associated with RE-induced (~67% 1RM) loss of abdominal fat and increased muscle density [18]. Skeletal muscle mass is typically regulated by the balance between muscle protein synthesis and muscle protein breakdown, where insulin can reduce the muscle protein breakdown, and thereby promote muscle protein turnover [68]. At a molecular level, increased muscle mass and muscle strength with RE are attributed to the increased muscle hypertrophy, which possibly occurs through PI3K-Akt-mTOR signaling cascades. Such molecular events may be associated with improved muscle substrate (glucose or fat) metabolism [3,69]. Resistance training (60–80% 1RM, twice/week) combined with AE for 12-months significantly reduced the HbA1c, blood glucose, body weight, and waist circumference in diabetic patients. These results were accompanied by increased skeletal muscle PPAR-γ and PPAR-α mRNA levels, which promote glucose and fat oxidation in skeletal muscle mitochondria of diabetic patients [70].

Furthermore, patients with T2D are characterized by reduced mitochondrial oxidative capacity per unit of muscle mass. However, RE alone or combination with AE reported to improve muscle mitochondrial oxidative capacity [71] and overall metabolic phenotype in patients with T2D [72]. In support of the ‘gene shifting’ hypothesis, 14-week RE at intensity 50–80% 1RM (3-times/week) reported to augment mitochondrial creatine kinase and cytochrome c oxidase and suppress oxidative DNA damage in elderly [73]. Additionally, 12-week resistance training at intensity 50–75% 1RM (twice/week) improved the cytosolic and mitochondrial antioxidant enzymes (superoxide dismutase, glutathione peroxidase) and subsequently reduced the oxidative stress in skeletal muscle of patients with T2D [74]. Another study showed 16-week RE intervention (60–85% 1RM, 3-times/week) significantly reduced the interleukin-6 and tumor necrosis factor-α, and changes in muscle strength was associated with response of pro-inflammatory cytokines in obese adults [75]. Since mitochondrial oxidative capacity, antioxidant status, and inflammation are intrinsically connected, RT-mediated improvements of those systems synergistically ameliorate HbA1c, insulin, and hyperglycemia in patients with T2D. These findings explain that resistance training with optimal intensity is the practical lifestyle intervention to treat T2D.

## 5. Conclusions

For the first time, our meta-analyses have provided additional evidence that high-intensity resistance exercise has greater beneficial effects than low-to-moderate-intensity in attenuation of elevated HbA1c and insulin levels in patients with T2D. Our results also emphasized the strong association of RE intensity with effective management of HbA1c and insulin. Nevertheless, whether these beneficial effects of RE can be achieved without significant reduction of blood glucose is still to be investigated. When it is necessary to prescribe RE therapy for patients with T2D, intensity should be the primary concern to accomplish the maximum benefits of RE, according to the patient’s physical fitness.

## Figures and Tables

**Table 1:** Characteristics of the trials included in the meta-analysis, presented in chronological order.

Study	Year, Country	Participants (M/F)		Mean Age (Y)	Diabetes Duration (Y)	Resistance Exercise Description	Intensity (% 1RM)	Repetitions	Sets	Frequency (t/wk)	Duration (wk)
		Exercise	Control								
Hsieh et al. [43]	2018, Taiwan/China	15 (5/10)	15 (6/9)	71.2 ± 4.3	RE:11 ± 7.8 C:13.9 ± 6.7	Chest press, shoulder press, bicep curl, hip abduction, standing hip flexion, leg press, standing calf raise, and abdominal crunch	75%	8–12	3	3	12
AminiLari et al. [45]	2017, Iran	15 (0/15)	15 (0/15)	45–60	At least 2	Leg extension, prone leg curl, abdominal crunch, biceps, triceps, and seated calf	50–55%	8	3	3	12
Mahdirejei et al. [37]	2014, Iran	9 (9/0)	9 (9/0)	48.5 ± 7.7	(nr)	Bench press, butterfly, lat pull-down, bicep curl, triceps extension, seated rowing, knee flexion, knee extension, and heel raise	50–80%	8–15	3	3	8
Mavros et al. [51]	2013, Australia	36 (nr)	48 (nr)	≥ 60	RE:7 ± 5 C:9 ± 7	Seated row, chest press, leg press, knee extension, hip flexion, hip extension, and hip abduction	80%	8	2–3	3	48
Hameed et al. [52]	2012, India	24 (18/6)	24 (17/7)	45 ± 4.1	> 0.5	Supine bench press, leg press, lateral pull, leg extension, and seated bicep curls	65–70%	10	3	2–3	8
Kadoglou et al. [41]	2012, Greece	23 (7/16)	24 (5/19)	61.3 ± 2	RE:6 ± 2.8 C:5.6 ± 1.9	Seated leg press, knee extension, knee flexion, chest press, lat pull-down, overhead press, bicep curl, and tricep extension	60–80%	6–8	2~3	3	12
Oliveira et al. [49]	2012, Brazil	10 (4/6)	12 (4/8)	54 ± 8.9	RE:7.7 ± 4 C:5.2 ± 3.5	Leg press, bench press, lat pull-down, seated rowing, shoulder press, abdominal curls, and knees curls	67–80%	15	4	3	12
Yavari et al. [46]	2012, Iran	20 (nr)	20 (nr)	51.5 ± 6.3	> 1	Bench press, seated row, shoulder press, chest press, lateral pull-down, abdominal crunches, leg press, leg extension, tricep pushdown, and seated bicep curls	75–80%	8–10	3	3	52
Kwon et al. [35]	2011, Korea	12 (0/12)	15 (0/15)	57 ± 6.8	RE:4.6 ± 2.7 C:4.9 ± 4.7	Curls, tricep extensions, upright rows, shoulder chest press, and seated rows. Core exercises included trunk side bends, leg press, hip flexions, leg flexions, and leg extensions	40–50%	10–15	3	3	12
Church et al. [42]	2010, America	73 (30/43)	41 (13/28)	55.8 ± 8.7	RE:7.2 ± 5.5 C:7.2 ± 5.2	2 sets of 4 upper body exercises (bench press, seated row, shoulder press, and pulldown), 3 sets of 3 leg exercises (leg press, extension, and flexion) and 2 sets each of abdominal crunches and back extensions	67%	10–12	2–3	3	36
Hazley et al. [20]	2010, England	6 (3/3)	6 (4/2)	53 ± 9	(nr)	Leg press, chest press, leg curl, leg extension, latissimus dorsi pull-down, press up, seated row, sit up, and bicep curl	50–60%	15	1–2	3.5	8
Ku et al. [47]	2010, Korea	13 (0/13)	16 (0/16)	55.7 ± 6.2	RE:5.7 ± 4.8 C:5.8 ± 6	Bicep curl, tricep extension, upright row, shoulder chest press, trunk side bending, seated row, leg press, hip flexion, leg flexion, and leg extension	40–50%	15–20	3	5	12
Plotnikoff et al. [53]	2010, Canada	27 (8/19)	21 (8/13)	55 ± 12	(nr)	Squats, seated row, chest press, shoulder press, lunges, lateral pull-down, standing tricep extension, standing pulley abdominal twists, bicep curl, tricep press, reverse rhomboid flies, lateral pulley deltoid raise, and pulley abdominal curls	50–85%	8–12	2–3	3	16
Wycherley et al. [33]	2010, Australia	17 (nr)	16 (nr)	56 ± 7.5	(nr)	Leg press, knee extension, chest press, shoulder press, lat pull down, seated row, tricep press, and sit-ups	70–85%	8–12	2	3	16
Arora et al. [38]	2009, India	10 (4/6)	10 (6/4)	53.8 ± 8.8	RE:5.4 ± 1.5 C:5.2 ± 3.9	Groups-biceps, triceps, upper back, abdominals, knee flexors, and extensors	60–100%	10	3	2	8
Shenoy et al. [50]	2009, India	10 (4/6)	10 (6/4)	49.6 ± 5.2	RE:5.4 ± 1.5 C:5.2 ± 2.9	Bicep curls, tricep curls, front lateral pull down, back lateral pull-down, knee extension exercises on quadriceps table, hamstring curls using quadriceps table and abdominal curls	60–100%	10	3	2	16
Baum et al. [54]	2007, Germany	13 (nr)	13 (nr)	62.9 ± 7.3	(nr)	Leg extension, seated leg flexion, leg press, seated calf raises, lat pulley, horizontal chest press, butterfly, and rowing	70–80%	10–12	1–3	3	12
Brooks et al. [40]	2007, America	31 (21/10)	31 (19/12)	66 ± 2	RE:8 ± 1 C:11 ± 1	Upper back, chest press, leg press, knee extension, and flexion	60–80%	8	3	3	16
Sigal et al. [36]	2007, Canada	64 (40/24)	63 (41/22)	54.7 ± 7.5	RE:6.1 ± 4.7 C:5.0 ± 4.5	Group A: Abdominal crunches, seated row, seated biceps curls, supine bench press, leg press, shoulder press, leg extension. Group B: abdominal crunches, lateral pulldown, triceps push-down, sitting chest press, leg press, upright row, leg curls	80%	7–9	2–3	3	26
Gordon et al. [39]	2006, America	15 (7/8)	15 (8/7)	67 ± 7	RE:9 ± 2 C:12 ± 3	Knee extension, chest press, leg curl, upper back and leg press	60–80%	8	3	3	16
Baldi and Snowling [44]	2003, New Zealand	9 (9/0)	9 (9/0)	47.9	> 3	Ten exercises involving major muscle groups in the upper and low body	65–75%	12	2	3	10
Dunstan et al. [34]	1998, Australia	11 (8/3)	10 (5/5)	51	RE:5.3 ± 1.4 C:5.1 ± 1.2	Leg extension, bench press, leg curl, dumbbell bicep curls, behind neck pulldown, calf raise, dumbbell overhead press, seated rowing, forearm extension using pulley (triceps), and abdominal curls	50–75%	10–15	3	3	8
Ishii et al. [19]	1998, Japan	9 (9/0)	8 (8/0)	46.8 ± 8.9	(nr)	Arm curls, military press, push-ups, squats, knee extensions, heel raises, back extensions, bent knee sit-ups, and upright rowing. Back extensions, push-ups, and bent knee sit-ups	40–50%	10–20	2	5	6
Honkola et al. [48]	1997 Finland	18 (12/6)	20 (5/15)	62 ± 2	RE:8 ± 2 C:8 ± 2	Thigh flexors and extensors, trunk flexors and extensors, upper arm muscles	65–67%	12–15	2	2	20

M/F, male/female; Y, years; 1RM, one-repetition maximum; t/wk, times/week; RE, resistance exercise; C, control; nr, not reported.
